# Emotions in green: unveiling the paradox of green human resource management in shaping employee conduct

**DOI:** 10.3389/fpsyg.2025.1699701

**Published:** 2025-12-11

**Authors:** Xueling Wang, Qingjin Wang, Jinlei Hao, Bolin Niu, Wenjie Xu, Yuhan Liu

**Affiliations:** ^1^Business School of Qingdao University, Qingdao, China; ^2^Business School of Lanzhou University of Finance and Economics, Lanzhou, China

**Keywords:** green human resource management, emotions, cognitive appraisal theory, green advocacy behavior, green value, workplace cheating behavior

## Abstract

This study examines the paradoxical dual effects of Green Human Resource Management (GHRM) in the hospitality industry. While prior comparative research has shed light on its positive impacts on organizations, the exploration of its potential adverse effects, particularly the reasons behind its paradoxical effects, is less developed. Grounded in the cognitive appraisal theory of emotion, this research analyze 407 valid survey responses from China to investigate the conditions under which GHRM leads to both benefical and detrimental organizational effects. The findings reveal that for employees with high green personal values, GHRM triggers positive emotions, thereby promoting organizational green advocacy behaviors. Conversely, for employees with lower green values, it induces negative emotions, which in turn increase workplace cheating behaviors. Theoretically, this study advances the understanding of the paradoxical nature of GHRM, particularly within the context of the hospiality sector. Practically, it offers valuable insights for implementing green management in the industry, broadening the comprehension of how corporate green management in the industry, broading the comprehension of how corporate green initiatives interact with employee values. These insights can assist the hospitality sector in adopting GHRM more effectively, enhacing environmental performance, and responding to the growing environmental concerns of customers and stakeholders.

## 1 Introduction

In the contemporary landscape of the industries, the adoption of sustainable practices has become a cornerstone for success and reputation. This trend is encapsulated in the widespread implementation of green human resource management (GHRM), a strategy that integrates environmental considerations into human resource policies and practices (Fawehinmi et al., 2020; Shah et al., 2024). The impetus for this shift stems from a blend of internal motivations, such as a desire for social responsibility and financial benefits, and external pressures, including stakeholder demands and regulatory frameworks (Tanova and Bayighomog, 2022; Mehrajunnisa et al., 2022; Merli et al., 2019). GHRM is instrumental in encouraging employee participation in sustainable initiatives, thereby contributing to the broader environmental goals of the organization. Prominent hospitality chains like Hilton, Marriott, and Ramada exemplify this trend through their active engagement in environmental programs (Kim et al., 2019; Hilton, 2023). The widespread adoption of GHRM accentuates the criticality of academic research dedicated to exploring its impact.

The current discussion on GHRM highlights its diverse advantages, such as improved organizational performance and environmental sustainability, as noted by researchers like Tabrizi et al. (2023) and Darvishmotevali and Altinay (2022). These advantages include increased organizational sustainability, innovation in green initiatives, and higher employee job satisfaction (Ren et al., 2021; Darvishmotevali and Altinay, 2022; Pham et al., 2020). Yet, there are noted challenges in implementing GHRM, including resource limitations and knowledge gaps, as identified by Tanveer et al. (2023). Paradox theory, referenced in research by Guerci and Carollo (2016), introduces further complexity by suggesting that GHRM can lead to a range of outcomes, not all positive, thereby underscoring a critical gap in our understanding of employee roles in GHRM. Recent studies have further explored the paradoxical effects of GHRM. For example, Peng et al. (2025) found that GHRM increases both the benefits and costs of employees’ green behavior at home due to job stress. Another study by Ahmed et al. (2024) suggests that GHRM may have dual effects on employees’ organizational citizenship behavior for the environment (OCBE) through opposing mechanisms, such as environmental passion and emotional exhaustion. Similarly, Jia et al. (2025) identified a U-shaped relationship between the alignment of GHRM and organizational green innovation. In their study, the positive effects of GHRM initially increased, then decreased, and eventually recovered, contingent upon employee perceptions of uncertainty. This theory emphasizes the need for a more nuanced examination of the conflicting effects of GHRM, particularly why negative outcomes may arise despite good intentions. This idea is further supported by List and Momeni (2021) and Zhang et al. (2021), who found that workplace practices can have diverse effects due to individual psychological factors. While much research, such as that by Abualigah et al. (2023), Tabrizi et al. (2023), and Tu et al. (2023), focuses on GHRM’s influence on employee environmental sustainability, it often neglects the impact of individual differences. Thus, there’s a call for more in-depth research into how personal factors affect GHRM outcomes, including potential negative effects, to deepen our understanding of GHRM’s complex impact in organizational settings.

A few recent studies have started to investigate the role of employee traits in determining the effectiveness of GHRM (Tandon et al., 2023). This research direction is in harmony with McClelland’s idea that values, desires, and abilities significantly influence how individuals act and react in certain situations (McClelland, 1985). Our study aims to build on this by looking at how GHRM impacts employees, especially how their green values shape their reactions to GHRM efforts. Employing the cognitive appraisal theory of emotion, our research examines the possible two-fold impact of GHRM at work. This theory posits that emotions arise in uncertain situations without a clear trigger, and cognitive appraisal acts as a “necessary and sufficient” trigger for emotion (Lazarus, 1982, 1991; Roseman, 1996). Therefore, employees assess events concerning themselves and form emotional responses to these events, which then indirectly shape their behavior within the organization (Roseman, 1991; Roseman, 1996). From this perspective, we suggest that GHRM elicits positive feelings based on the “motive-consistent” principle in employees who highly value eco-friendly behaviors, thereby enhancing their advocacy for green practices at work. Conversely, for employees with low green values or “motive-inconsistent,” the company’s green efforts might be seen as conflicting with their interests, leading to negative emotions and a rise in self-serving actions, including deceptive behaviors at work (Mitchell et al., 2018). Our research aims to offer an in-depth understanding of GHRM’s influence on employee conduct, considering the variety of individual values and how they affect organizational outcomes.

This study contributes to the current GHRM and hospitality literature in three ways. First of all, we reveal the double-edged sword consequences of GHRM since most existing GHRM studies tend to assume that those green practices have a uniform effect in hotels (i.e., rising in employee eco-friendly behavior, increases in environmental performance, rise in green creativity), but are silent on considering the potential process of a negative effect in mind of not every employee is an advocate for the concept of going green (Ahmed et al., 2021; Ali et al., 2022; Farooq et al., 2022). Conflict could emerges between internal cognition and external requirement in the implementation of GHRM, which could lead to corresponding behaviors. In doing so, we fill the gap that explored the exhausting effect of GHRM on employees, discussing both constructive and counterproductive workplace behaviors, providing a new perspective for hospitality industries to implement the green management process. This nuanced approach can help in leading to more sustainable and effective green management practices in the hospitality industry.

Secondly, our research integrates the cognitive appraisal of emotion theory into GHRM studies, offering a novel perspective on how GHRM can influence diverse workplace behaviors through emotional pathways. While existing studies have focused on motivational, organizational, and social learning factors (Abualigah et al., 2023), we highlight the significant role of emotional reactions in shaping employee responses to GHRM. We address a notable gap in literature by focusing on the emotional aspects of employee interactions with environmental policies, proposing that managing these emotional responses is crucial for the success of GHRM strategies. The findings suggest that hospitality managers need to be aware of the diverse responses to GHRM and develop strategies that not only promote green practices but also address the emotional and psychological needs of their employees.

Thirdly, our research introduces an innovative angle by exploring the role of individual green values in shaping the impact of GHRM. This approach not only investigates how these values influence the effectiveness of GHRM but also sheds light on the root causes of conflicting result triggered by GHRM practices. Diverging from previous research that primarily focused on the influence of factors such as leadership, organizational culture, and psychological climate on employee behavior (Tu et al., 2023; Shahzad et al., 2023; Naz et al., 2023), our study pioneers a new direction by evaluating the effect of personal values on the effectiveness of GHRM initiatives. The core aim of our research is to identify specific employee profiles that are more likely to demonstrate either supportive or detrimental behaviors in response to GHRM initiatives. By addressing this gap, our study responds to recent scholarly calls for research that explores the interplay between individual characteristics and GHRM outcomes, as highlighted in recent literature (see Bahuguna et al., 2023; Tandon et al., 2023). Furthermore, our study intends to offer actionable insights for HR practitioners. By identifying the traits of employees who positively engage with GHRM, HR professionals can tailor their approaches to enhance the acceptance and effectiveness of these initiatives. This could involve targeted training programs, personalized communication strategies, and incentives aligned with individual environmental values. Hence, our research not only contributes to the academic discourse on GHRM but also offers practical implications for organizations striving to integrate sustainability into their human resource practices.

## 2 Theoretical background and hypotheses

### 2.1 GHRM on employees’ behavior

Green human resource management encompasses the adoption of eco-friendly policies to influence employee behavior (see Aftab et al., 2023; Bahuguna et al., 2023). Specifically, GHRM initiatives increase awareness of environmental concerns, encouraging environmentally friendly actions, organizational citizenship behaviors focused on the environment, and innovative green solutions (Naz et al., 2023; Li et al., 2023; Abualigah et al., 2023; Murillo-Ramos et al., 2023). Additionally, studies have shown a direct positive link between GHRM and several organizational behaviors like higher job commitment, reduced staff turnover, and greater job satisfaction (Chaudhary, 2020; Shafaei et al., 2020; Shoaib et al., 2021). Recent studies have explored the emotional dynamics associated with Green Human Resource Management (GHRM) and its effects on employee behavior. For example, GHRM has been found to enhance employees’ subjective well-being by fostering pro-environmental behavior (Gyensare et al., 2024). Additionally, GHRM positively influences employees’ workplace green behavior through increased environmental commitment. However, it has also been shown to reduce voluntary workplace green behavior (VWGB) by contributing to emotional exhaustion (Yuan et al., 2024). Furthermore, several studies have explored GHRM’s effects on employee behavior from a geographical perspective. For example, Alshahrani and Iqbal (2024) conducted a study in Saudi Arabia and the broader Middle East to investigate how GHRM influences organizational pride and employee identification. Additionally, research in North India has examined the relationship between GHRM and work engagement (Gupta and Jangra, 2024), while studies in China have explored the role of GHRM in fostering employee green activism (Usman et al., 2025). These findings highlight the critical role employees play in adopting and integrating these green policies. Therefore, it’s vital for employees to evaluate and understand the impact of these green initiatives.

### 2.2 Cognitive appraisal theory of emotion

Roseman (1996) first introduced the concept of cognitive appraisal theory of emotion to the field of organizational behavior; growing awareness of employees’ behavior has driven a line of research on how organizational practice triggers employees’ emotions and emotion feedback.(Roseman, 1996). The cognitive appraisal theory of emotion emphasizes the close connection between emotion and an individual’s subjective cognition of events, which indicates that emotion is an individual’s evaluation response to an event (Roseman, 1996). According to the theory, the generation of emotions depends on an individual’s cognitive evaluation of a specific event and individuals appraisals of the event based on its importance, consistency, and significance of the event (Lazarus, 1982). Then, different individuals have different determining factors in interpreting self-involved stimuli based on their subjective norms, invoking their different emotional reactions toward a same stimuli (Lazarus,1982; Roseman, 1996). In the workplace, job-related activities (e.g., job duties, communication, training) are important stimuli that influence workers’ behavior and perception, triggers different reaction. Accordingly, drawing on insight of the cognitive appraisal theory of emotion, GHRM practice requires employees’ cognitive involvement regarding the process and appraisal of the self-relevant concept of the event, and then the process triggers either a positive or negative emotion toward the practice based on personal value and attitudes. When the company launches GHRM policies, employees appraise the practices based on their norms and interpret different perceptions regarding those activities. Thereby, individual behavior changes based on their cognition causes by the events stimulation.

### 2.3 Positive emotion pathway on green advocacy behavior and the moderating role of personal green value

Drawing from the established theory, positive emotions in the workplace arise when there is an alignment between employees’ personal values and their organizational behaviors, benefiting both the individual and the organization (Edwards and Cable, 2009; Roseman, 1996). Specifically, in the context of green human resource management (GHRM), an employee’s green values play a crucial role in shaping their perception and motivation towards environmentally friendly behaviors (Steg et al., 2005). We suggest that GHRM is more likely to elicit positive emotions in employees who hold strong green values. The engagement of employees in HRM practices involves a cognitive process where they appraise the relevance of these practices to their personal values, leading to either positive or negative emotional responses. Positive emotions, such as pride, are typically experienced when individuals perceive a stimulus as aligning with their personal motivations (Roseman, 1996; Lazarus and Smith, 1988).

In the case of GHRM, an employee’s personal green value acts as a key motivational factor, influencing their attitudes towards eco-friendly practices. This value system encompasses personal norms and behaviors that favor environmental sustainability (Becerra et al., 2023). Employees with strong green values experience a sense of ‘self-consistent appetitive motives’ towards green activities. This ‘appetitive’ state is characterized by a pursuit of rewards, evoking positive emotions like joy, liking, and pride (Roseman, 1996). Therefore, employees who share the organization’s green values are more likely to experience positive emotions in response to GHRM initiatives. This is consistent with previous research indicating that value congruence between employees and their leaders is crucial for fostering positive emotions in the workplace (Edwards and Cable, 2009). Conversely, employees with lower green values may not perceive GHRM activities as motivationally consistent, potentially diminishing their positive emotional response to these initiatives.

Further, we propose that employees who hold stronger environmentally friendly values, as opposed to those with lower values, are more likely to engage in pro-environmental advocacy behavior at the workplace, driven by a heightened sense of positivity stemming from GHRM practices. Such green advocacy involves sharing environmental knowledge among peers (Kim et al., 2017). Positive reactions to GHRM spur employees to deepen their dedication to sustainable practices and inspire similar behaviors in coworkers. Positive emotions typically foster prosocial and group-oriented behaviors (Piff et al., 2015), and research indicates they increase pro-organizational actions beneficial to the company (Sturm et al., 2022). For example, Boğan and Dedeoğlu (2020) noted that work-proud individuals often exhibit organizational citizenship behavior. Hence, green advocacy, as an aspect of pro-organizational behavior, reflects employees’ active promotion of eco-friendly actions and their encouragement of coworker participation (Kim et al., 2017). Further, recent studies suggest that employees with a strong personal environmental stance are more proactive, suggesting improvements for the workplace (Palmié et al., 2023).

Positive feelings also enhance employees’ favorable views of their workplace, fostering a connection with the organization when its green actions resonate with their values. This motivates them towards personal achievements and fulfilling job duties. Those with high self-achievement are more likely to adopt workplace eco-friendly behaviors. Additionally, positive emotions are crucial for increasing job satisfaction and self-motivation (Todorova et al., 2014), leading to greater commitment and loyalty among employees who value green initiatives, which, in turn, encourages behaviors aligning with the company’s objectives (Meyer et al., 1989). Thus, GHRM-influenced employees with positive emotions are more disposed to support and contribute to green initiatives.

Moreover, positive emotions enhance interpersonal relationships at work (Ashkanasy and Dorris, 2017). When employees respond positively to green concepts, it inspires colleagues to adopt similar behaviors to experience these positive effects. Additionally, encouraging feedback from peers motivates further green advocacy. Therefore, we propose that positive emotions significantly boost employee green advocacy in the workplace. We hypothesize that the positive indirect impact of GHRM on employee green advocacy, mediated through positive emotions, is more pronounced in employees with high green values compared to those with lower values. Hence, we propose:

Hypothesis 1: The relationship between GHRM and positive emotional response is stronger in employees with high green values (versus low).

Hypothesis 2: The positive indirect effect of GHRM on employee green advocacy behavior as mediated by positive emotion is stronger when employees’ green value is high (versus low).

### 2.4 Negative emotion pathway of workplace cheating behavior and the moderating role of personal green value

While GHRM often promotes positive feelings and behaviors among some employees, it’s essential to understand that personal reactions can differ greatly due to individual mental frameworks (Roseman, 1996; McCauley et al., 2006). Specifically, we suggest that employees with lesser eco-friendly values might respond with a “motive-inconsistent” attitude to GHRM, sparking negative emotions (Roseman, 1996).

Firstly, according to the theory of cognitive appraisal of emotion, negative emotions like dislike and anger often arise when individuals appraise an event as both incongruent with their personal motives and outside of their volitional control (Roseman, 1996). GHRM involves engaging in eco-friendly activities like green training, which adds to their existing workload. This additional burden can cause negative feelings, particularly in those less committed to green values. Furthermore, GHRM can contribute to increased work-related stress, especially for employees who perceive green practices as insignificant. Negative emotions can intensify when conflicts arise between employees’ regular work duties and GHRM initiatives. For example, when a project is time-sensitive, and employees are required to dedicate extra time to participate in GHRM activities like green education or meetings, work stress may escalate. Work stress is a significant trigger for negative employee emotions, particularly in those with lower green values (i.e., motive-inconsistent individuals) (Diefendorff et al., 2008; Rubel et al., 2021).

Further, the inclusion of green performance appraisal as a component of GHRM can have repercussions. This appraisal system is used to evaluate employees’ green behaviors in the company, with the aim of promoting pro-environmental behavior (Shah, 2019). Individuals evaluate events related to themselves based on their “motivational state,” which can lead to either “appetitive” or “aversive” cognitions (Roseman, 1991; Roseman, 1996). For employees with low green values, “aversive” cognition may emerge as they seek to avoid potential punishment, resulting in negative emotions such as distress, frustration, and dislike (Roseman, 1991). This situation can worsen for employees who are not inclined toward green practices but face penalties for non-compliance. For example, the GHRM’s reward system may offer bonuses or impose fines based on employees’ green performance. Employees who are personally disinclined towards green behavior are not only required to engage in GHRM but also face potential losses for failing to comply. Lastly, employees less inclined towards eco-friendly values might face social stress under GHRM, leading to a range of negative emotions. They might feel isolated or stressed about not meeting the company’s green expectations, resulting in stronger negative responses to GHRM compared to their more eco-conscious peers.

We hypothesize that such emotional responses may give rise to workplace cheating behavior. Rooted in humans’ natural inclination to protect themselves, people instinctively engage their self-regulatory systems to cope with negative feelings. According to Thayer et al. (1994), those overwhelmed by such emotions are more likely to act unethically at work due to this self-preservation instinct. Mitchell et al. (2018) suggest that individuals under the sway of negative feelings tend to prioritize their own needs, which may lead to dishonest actions. Cheating at work includes any unethical behavior that favors personal gain over professional ethics, like causing conflicts, being absent without good reason, or making unwarranted complaints (Kaptein, 2008; Yue et al., 2023). When influenced by negative emotions, employees are more likely to divert their attention away from self-control compared to those who are not experiencing such emotions (Chester et al., 2016). Research in emotional psychology indicates that those experiencing negative emotions are more likely to behave destructively at work (Feinberg et al., 2020). Furthermore, people prone to feelings like anger and anxiety are likelier to act selfishly, increasing their propensity for workplace deceit (Hillebrandt and Barclay, 2022). Negative feelings can push employees to put their interests before the company’s, leading to ethical disconnection (Fida et al., 2015). In tough situations, workers might activate their self-defense mechanisms to alleviate stress, disregarding whether their actions align with company goals (Miller, 1999).

Additionally, GHRM is mandatory daily, regardless of personal beliefs. This mismatch between personal values and GHRM requirements can lead to absenteeism and deceit among employees who feel disconnected. Even those who do not personally support green initiatives must comply with corporate policies, potentially leading them to circumvent these obligations. Such misalignment might prompt employees to reduce their green efforts and resort to deceitful behaviors in the workplace.

Lastly, those with weaker green values might feel pressured by higher-ups or peers with stronger green commitments due to GHRM mandates. This pressure can drive them to dishonest behaviors, fearing negative consequences for not adhering to company policies. They might engage in surface acting or impression management, presenting a pro-environmental facade while internally rejecting these values. As green values are part of one’s ethical code, those with lower green values might disconnect more from moral standards, making them more likely to engage in unethical workplace actions to boost their image.

Hence, we propose:

Hypothesis 3: The relationship between GHRM and negative emotion is stronger when the employee's green value is low (versus high).

**Figure 1:**
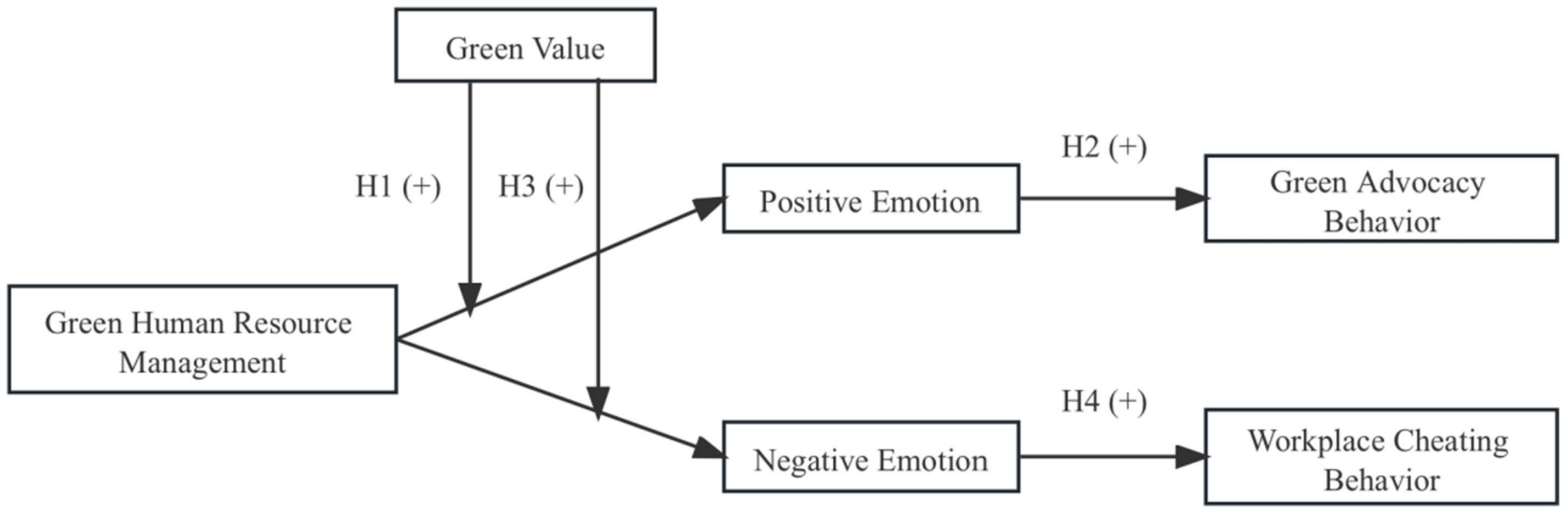
Theoretical model.

Hypothesis 4: The positive indirect effect of GHRM on workplace cheating behavior as mediated by negative emotion is stronger when employees’ green value is low (versus high) (Figure 1).

## 3 Methodology

### 3.1 Procedure and data collection

A total of 1,000 participants were selected from hotels in Qingdao, China, that implemented Green Human Resource Management (GHRM) practices. This sample size was chosen to ensure sufficient statistical power for detecting meaningful differences and relationships within the data. As noted by Cohen (1992), a sample size of 1,000 is typically adequate to yield reliable and generalizable results. Additionally, a sample of this size provides a robust foundation for minimizing potential sampling bias and enhances the external validity of the findings. The choice of 1,000 participants is consistent with the sample sizes employed in similar studies within the hospitality and human resource management literature, such as those by Chen et al. (2020) and Wang et al. (2019). Data collection was conducted in Qingdao, which facilitated direct interaction between our research team and the participants. This face-to-face approach enabled us to clearly explain the study, thereby minimizing the biases commonly associated with online surveys. Moreover, recent research has identified Qingdao as a key case study for analyzing the spatial patterns of tourist flows, further underscoring the significance of this location within the context of tourism and hospitality (Mou et al., 2020). To identify potential sample hotels, our research team collaborated with the corresponding author and engaged with the Qingdao Hospitality Chamber of Commerce. The initial questionnaire was sent via email for review and discussion. After outlining the study’s objectives to the Chamber’s coordinator, we requested their assistance in contacting the participating hotels. We provided a document containing cover letters and the survey instrument for their evaluation. Following this, the coordinator shared our contact details and research materials within the Chamber’s group chat.

Following approval from participating hotels, we selected the final sample and commenced data collection with the support of the hotels’ HR departments. The sampling procedure is best described as a quasi-random method. We randomly selected participants from the frontline employees’ roster, choosing every third person (e.g., 3rd, 6th, 9th). If any selected employee was unavailable, we replaced them with other convenience samples.

Data collection occurred from November 11, 2024, to December 11, 2024. Prior to the main study, we conducted pilot testing with 10 employees to address any issues and verify the content’s validity. Their feedback was incorporated into the final version of our instrument. We distributed our questionnaires in three phases. In the initial phase, we briefly described our research and its aim to improve the company’s HR management. We assured employees of confidentiality, addressed their queries, and obtained their informed consent, noting they could withdraw at any time. Participants were organized into 10 groups (*N* = 50). In this phase, participants could receive a red packet via QR code or a ¥5 gift card. Each questionnaire had a unique identification number for tracking across phases. We gathered GHRM and demographic data, achieving an 89% return rate (*N* = 890) due to effective communication and engagement. Two weeks later, the second phase focused on positive and negative emotions. Participants were instructed to write their assigned number from the first phase on the questionnaire to maintain consistency. Each received an ¥8 gift card, with a 70% return rate (*N* = 623). After another 2 weeks, we explored workplace cheating behavior and coworker green advocacy, offering a ¥10 red packet, yielding a 66% return rate. In the final phase, due to time constraints, we mailed the questionnaires to the HR office for collection, which may explain the lower return rate. Ultimately, we received 415 questionnaires, discarding those with clear errors, resulting in 407 valid responses.

### 3.2 Measurement

We utilized SPSS 26 and Mplus 8.4 for all data analysis. Factors like age, gender, education, hotel type, tenure, and income were accounted for due to their potential impact on employee attitudes. Additionally, G*Power was employed to determine the ideal study sample size (Effect size = 0.08, power = 0.95, target sample size = 304). Results were gauged using a six-point Likert scale ranging from 1 to 6. English survey questions were translated to Chinese as per Brislin (1970) methodology. The surveys, which included well-established questions, were translated by professional translators to ensure clarity.

We use the scale developed by Dumont et al. (2017) to test employees’ perceptions regarding the company’s GHRM. Other scholars have also used the scale (e.g., Rubel et al., 2021; Farooq et al., 2022). Example items include “My company sets green goals for its employees.” The Cronbach’s alpha is 0.926.

We tested the positive emotion toward the GHRM and were measured with reference to a scale developed by Watson et al. (1988). We selected 10 items that represent positive emotion in our questionnaires. Example items include “In the last 2 weeks, how often were the emotions “inspired” experienced at work “. The scale also been implemented by other scholar such as Avey et al. (2008). Cronbach’s alpha is 0. 953.

The concept of negative emotion was tested from a scale applied by Liu et al. (2007). The scale has been applied by serval other scholars in the field of psychology and business management (Nixon et al., 2011; Dawson et al., 2016). Example items cover “In the last 2 weeks, how often were the emotion “anger” experienced at work?” The Cronbach’s alpha is 0.91.

Items regarding green advocacy behavior were tested with a scale developed by Kim et al. (2017). Example items of the questionnaire cover “I try to convince my group members to reduce, reuse, and recycle office supplies in the workplace.” The Cronbach’s alpha is 0.928.

Workplace cheating behavior was measured with a scale expanded by Mitchell et al. (2018). Example items cover “Made it look like you were working when you were not.” The Cronbach’s alpha is 0.947.

**Figure 2:**
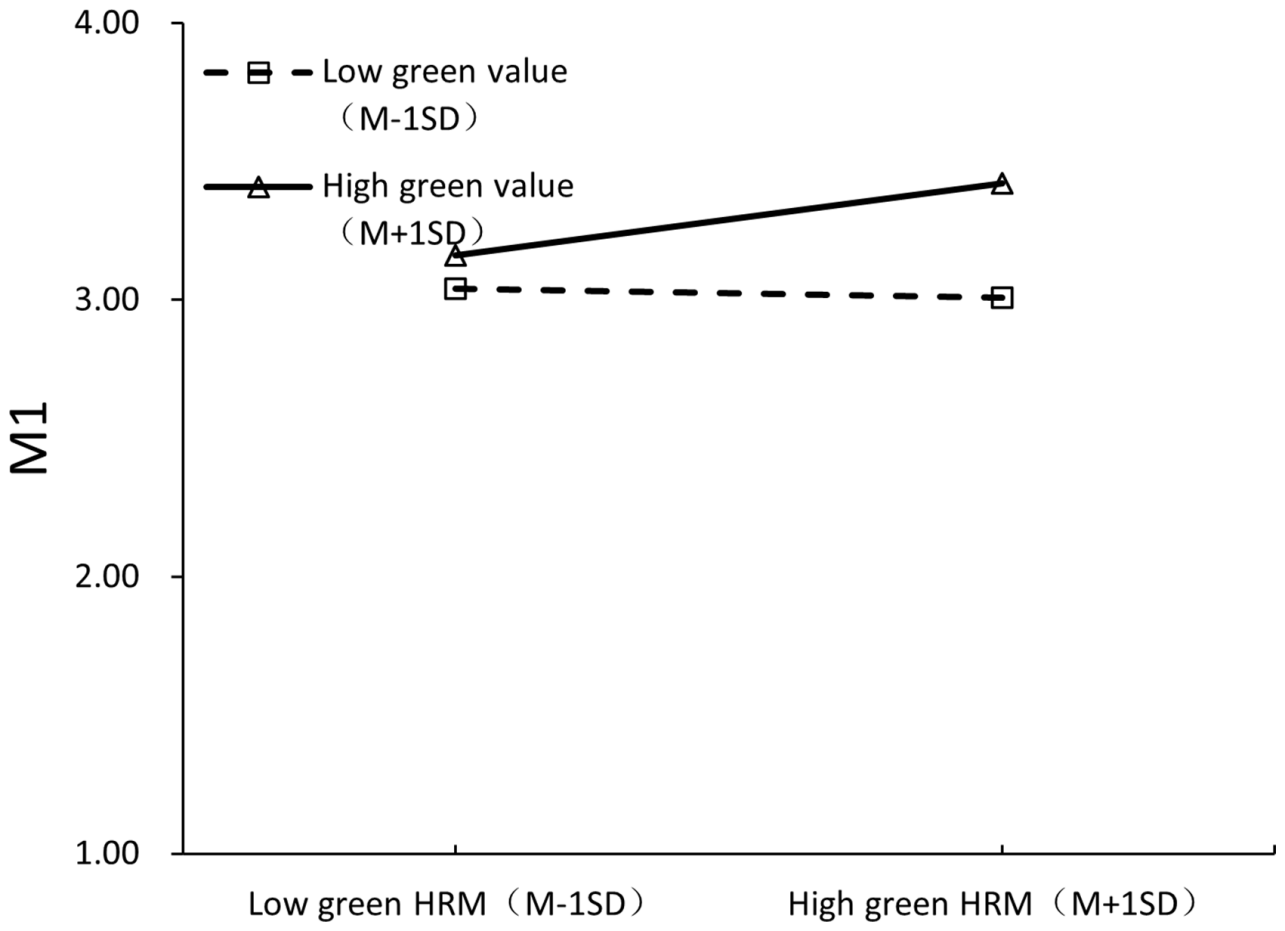
The interactive effect of GHRM and green value on positive emotion.

The personal green value was tested using a scale that has been developed by Steg et al. (2005). The scale has been used by serval other scholars as well (e.g., Tu et al., 2022; Lin and Yang, 2023). Example items cover “I feel personally obliged to save as much energy as possible.” The Cronbach’s alpha is 0.917 (Figure 2).

## 4 Result

### 4.1 Data analysis

To ascertain the model’s discriminant validity, this research utilized Mplus 8.4 for confirmatory factor analysis. Results presented in Table 1 indicate the six-factor model meets the established criteria and surpasses various alternative models. It was found that the indicators of the six-factor model were in line with the standard (CMID/DF = 1.488, RMSEA = 0.035, CFI = 0.972, TLI = 0.969) and are significantly better than other types of alternative models such as the five-factor model with a combination of green value and workplace cheating behavior (CMID/DF = 4.18, RMSEA = 0.088, CFI = 0.813, TLI = 0.801), and the one-factor model, with (CMID/DF = 11.499, RMSEA = 0.161, CFI = 0.375, TLI = 0.341). Furthermore, the study employed Harman’s single-factor test, analyzing all questionnaire items through unrotated factor analysis. The first principal component explained 15.6% of the variance, considerably less than the total variance explained (70.9%).

**Table 1:** Confirmatory factor analysis results for measures of all variables.

Model and structure	CMIN	df	CMIN/df	RMSEA	CFI	TLI
Six-factor model	1079.018	725	1.488	0.035	0.972	0.969
Five-factor model	3051.300	730	4.18	0.088	0.813	0.801
Four-factor model	3404.359	734	4.638	0.095	0.785	0.772
Three-factor model	5018.456	737	6.809	0.119	0.656	0.636
Two-factor model	7120.117	739	9.634	0.146	0.487	0.458
One-factor model	8508.988	740	11.499	0.161	0.375	0.341
Common method bias model	1090.323	686	1.589	0.038	0.967	0.963

Six-factor model: hypothesized model. Five-factor model: green value and workplace cheating behavior combined. Four-factor model: green advocacy behavior, workplace cheating behavior and green value combined. Three-factor model: negative emotion, green advocacy behavior, workplace cheating behavior and green value combined. Two-factor model: positive emotion, negative emotion, green advocacy behavior, workplace cheating behavior and green value combined. One-factor model: all variables combined.

To further substantiate these findings, the present study implemented the latent marker variable approach by introducing an independent latent factor for common method bias into the established six-factor model. This additional factor was specified to load on all observed indicators, following which a confirmatory factor analysis was reconducted. A comparison of model fit indices before and after incorporating the method factor revealed only negligible improvements: ΔCMIN = 11.305, ΔDf = 39, ΔCMIN/Df = 0.101, ΔRMSEA = 0.003, ΔCFI = 0.005, ΔTLI = 0.006 (as shown in Table 1). These observed differences are well below established thresholds for meaningful model improvement, indicating that the common method bias factor contributes minimally to the model’s overall explanatory power and does not substantively account for the covariance among the core constructs. Consequently, the findings suggest common method bias has a minimal effect on the study.

The convergent validity of the measures was evaluated, reflecting the extent to which the observed indicators effectively capture their intended theoretical construct. This evaluation, consistent with established methodological practice, was based on three key metrics: standardized factor loadings, Average Variance Extracted (AVE), and Composite Reliability (CR). The generally accepted thresholds for these measures are: standardized factor loadings > 0.50, AVE > 0.50, and CR > 0.70. As presented in Table 2, all constructs meet or exceed these criteria. The comprehensive results confirm that all measurement indicators strongly represent their respective theoretical constructs, thereby establishing adequate convergent validity for the measurement model.

**Table 2:** Validity test result.

Latent variable	Observed variable	Factor loading	AVE	CR	Square root of AVE
GHRM	Q1	0.887	0.860	0.974	0.927
	Q2	0.932			
	Q3	0.931			
	Q4	0.941			
	Q5	0.932			
	Q6	0.939			
Positive emotion	Q31	0.848	0.746	0.967	0.864
	Q32	0.849			
	Q33	0.881			
	Q34	0.850			
	Q35	0.858			
	Q36	0.891			
	Q37	0.854			
	Q38	0.871			
	Q39	0.858			
	Q40	0.873			
Negative emotion	Q17	0.903	0.821	0.958	0.906
	Q18	0.900			
	Q19	0.902			
	Q20	0.911			
	Q21	0.915			
Green advocacy behavior	Q7	0.822	0.736	0.893	0.858
	Q8	0.877			
	Q9	0.874			
Workplace cheating behavior	Q10	0.833	0.719	0.947	0.848
	Q11	0.844			
	Q12	0.799			
	Q13	0.867			
	Q14	0.853			
	Q15	0.881			
	Q16	0.855			
Green value	Q22	0.859	0.685	0.951	0.828
	Q23	0.865			
	Q24	0.815			
	Q25	0.884			
	Q26	0.871			
	Q27	0.820			
	Q28	0.615			
	Q29	0.806			
	Q30	0.880			

### 4.2 Descriptive statistics

The descriptive statistics are in Table 3.

**Table 3:** Descriptive statistic.

Variable	Mean	SD	1	2	3	4	5	6	7	8	9	10	11	12
Tenure	2.57	1.114	1											
Age	2	0.823	0.563**	1										
Salary	2.94	1.395	0.222**	−0.084	1									
Gender	0.62	0.486	−0.098*	0.016	−0.276**	1								
Job position	1.46	0.499	−0.149**	0.027	−0.425**	0.150**	1							
Educational level	3.34	1.111	0.022	−0.39**	0.570**	−0.193**	−0.28**	1						
GHRM	3.773	0.976	0.116*	0.194**	0.002	0.021	−0.053	−0.234**	1					
Positive emotion	3.193	0.517	0.072	−0.013	0.006	−0.07	−0.051	0	0.209**	1				
Negative emotion	1.833	0.986	−0.07	−0.115*	0.081	−0.04	−0.047	0.217**	−0.169**	−0.2**	1			
Green advocacy behavior	3.939	0.861	0.078	0.145**	−0.025	0.021	−0.058	−0.211**	0.614**	0.294**	−0.265**	1		
Workplace cheating behavior	2.138	0.914	−0.103*	−0.179**	0.108*	−0.096	−0.113*	0.257**	−0.232**	−0.073	0.483**	−0.265**	1	
Green value	4.12	0.713	0.067	0.122*	−0.018	0.016	−0.029	−0.173**	0.557**	0.281**	−0.355**	0.526**	−0.355**	1

*N* = 407, *p < 0.05, **p < 0.01.

### 4.3 Hypothesis testing

**Figure 3:**
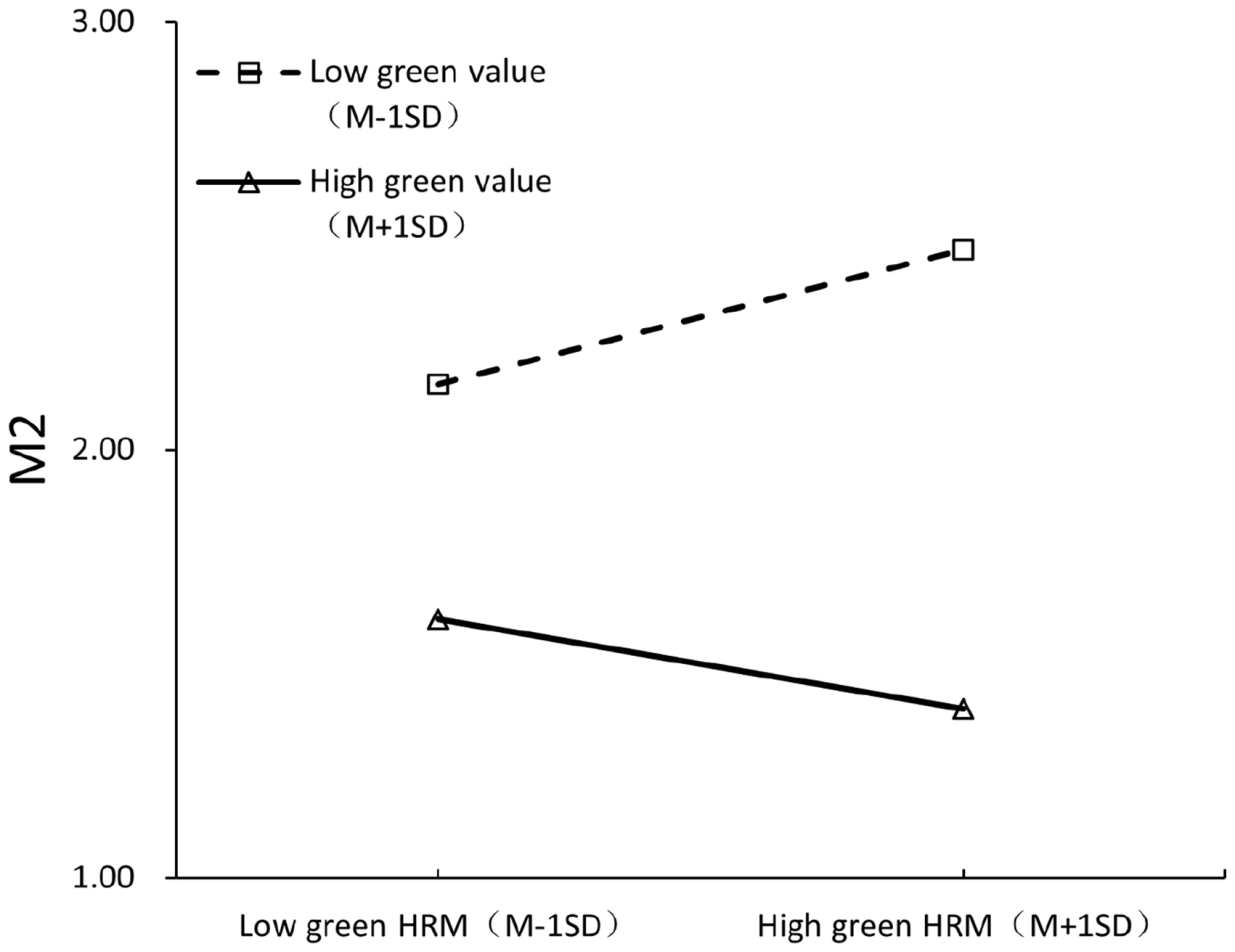
The interactive effect of GHRM and green value on negative emotion.

Using Mplus 8.4, a path analysis was performed with a maximum likelihood estimator (bootstrap = 5,000). To examine differences between individuals with high and low levels of green value, we created distinct groups using a ± 1 standard deviation (SD) split from the mean. Participants scoring more than 1 SD above the mean (>M + 1 SD) comprised the high green value, while those scoring more than 1 SD below the mean (< M - 1 SD) comprised the low green value. In substantive terms, scores > + 1 SD represent employee with a high green value while scores < −1 SD represent employees with a low green value. Participants scoring within ±1 SD of the mean were excluded from primary group comparisons to maximize distinction between the extreme groups. Hypothesis 1 posited that personal green values moderate the influence of GHRM on positive emotion, and this was confirmed (b = 0.141, *p* < 0.01). As detailed in Table 4, GHRM significantly boosts positive emotion (*b* = 0.075, *p* < 0.01), and the correlation between personal green value and positive emotion was also significant (*b* = 0.223, *p* < 0.001). Testing Hypothesis 1 revealed that GHRM’s positive impact on positive emotion is more pronounced when employees’ green values are high (*b* = 0.216, *p* < 0.001) rather than low (*b* = −0.067, *p* = 0.322), supporting the hypothesis. The results confirm that personal green values moderate the relationship between GHRM and positive emotions. When personal green values are high, GHRM has a significantly stronger positive effect on emotion, which supports the idea that employees who value sustainability are more positively influenced by green initiatives in the workplace. Additionally, Hypothesis 4 was validated, indicating a notable interactive effect between GHRM and personal green value on negative emotion (*b* = −0.227, *p* < 0.01; refer to Figure 3). The influence of GHRM on negative emotion is more substantial when employees’ green value is low (*b* = 0.27, *p* < 0.01) but not high (*b* = −0.184, *p* = 0.073), hence confirming Hypothesis 4. The findings demonstrate that the impact of GHRM on negative emotion is more pronounced when personal green values are low, suggesting that employees with low green values experience more negative emotions as a result of GHRM. The results also upheld Hypotheses 2 and 5, showing that positive emotion significantly promotes workplace green advocacy behaviors (*b* = 0.301, *p* < 0.001), and negative emotion considerably affects workplace cheating behaviors (*b* = 0.385, *p* < 0.001). Positive emotions drive workplace green advocacy behaviors, while negative emotions lead to workplace cheating behaviors. This is consistent with the hypothesis that emotions play a significant role in shaping workplace behaviors in response to GHRM.

**Table 4:** Path analysis results.

Variable	Positive emotion		Negative emotion		Employee green advocacy behavior		Workplace cheating behavior	
	*b*	*SE*	*b*	*SE*	*b*	*SE*	*b*	*SE*
Intercept	3.195***	0.192	1.696***	0.301	3.292***	0.373	1.657***	0.299
Controls								
Tenure	0.054	0.031	−0.066	0.054	−0.009	0.04	−0.045	0.046
Age	−0.079	0.044	0.046	0.078	0.012	0.063	−0.037	0.07
Salary	−0.027	0.023	−0.026	0.04	0.01	0.031	−0.018	0.039
Gender	−0.058	0.052	−0.054	0.099	0.023	0.074	−0.105	0.088
Position	−0.038	0.056	−0.034	0.096	−0.075	0.074	−0.147	0.083
Educational level	0.04	0.032	0.139*	0.055	−0.075	0.046	0.088	0.054
Focal variable								
GHRM	0.075**	0.036	0.043	0.065	0.485***	0.046	−0.119**	0.044
Personal green value	0.223***	0.053	−0.583***	0.091				
GHRM * Personal green value	0.141**	0.055	−0.227**	0.079				
Mediators								
Positive emotion					0.301***	0.081		
Negative emotion							0.385***	0.047
*R*-squared	0.156		0.196		0.414		0.279	

*N* = 407, *P < 0.05, **P < 0.01, ***P < 0.001.

Besides, our hypotheses 3 and 6 predict that personal green values indirectly influence workplace various behaviors in responding to the GHRM through different levels of emotion. The result confirmed our hypothesis 3 that GHRM had a positive indirect effect on coworker green advocacy behavior when employees’ personal value was high (*ab* = 0.65, SE = 0.027, 95% CI [0.023, 0.131]) compared with the low value (−0.02, SE = 0.023, 95% CI [−0.78, 0.013]). The difference (95% confidence interval) between high green value and low green value did not include zero (difference = 0.085, 95%CI [0.02, 0.201], see Table 4). Moreover, supporting hypothesis 6, GHRM had a positive indirect effect on workplace cheating behavior when employees’ personal green value was low (0.104, SE = 0.041, 95%CI [0.021, 0.185]) but not high (−0.071, SE = 0.04, 95% CI [−0.149, 0.008]). The 95% confidence interval in the difference of these effects did not include zero (−0.175, 95% CI [−0.295, −0.047]). The results confirm the indirect effects of GHRM on workplace behaviors through emotions. Specifically, GHRM enhances coworker green advocacy when personal green values are high and increases workplace cheating when personal green values are low, emphasizing the moderating role of personal values on the emotional outcomes and subsequent behaviors in the workplace.

As shown in Table 5, we further examine the indirect effect of GHRM on workplace behavior across different levels of personal value, the indirect impact of GHRM on coworker green advocacy behavior is stronger through positive emotion when personal green value is high (*b* = 0.065, 95% CI = [0.023, 0.131]) compare to low (*b* = −0.02, 95% CI = [−0.078, 0.013], n.s.). Alike, the indirect impact of GHRM on workplace cheating behavior is stronger through negative emotion when personal green value is low (*b* = 0.104, 95% CI = [0.021, 0.185]) compare to low (*b* = −0.071, 95% CI = [−0.149, 0.008], n.s.).

**Table 5:** Summary of indirect effect of GHRM on workplace behavior through different levels of value.

Outcomes	Mediator	Level of moderator	Conditional indirect effect	Lower bound	Upper bound
Employee green advocacy behavior	Positive emotions	High green value	0.065**	0.023	0.131
		Low green value	−0.02	−0.078	0.013
		Difference	0.085	0.02	0.201
Workplace cheating behavior	Negative emotions	High green value	−0.071	−0.149	0.008
		Low green value	0.104**	0.021	0.185
		Difference	−0.175**	−0.295	−0.047

*N* = 407, **P < 0.01.

In interpreting these effect sizes, it is important to consider their practical significance beyond statistical significance. The point estimate of 0.065 indicates that for every one-unit increase in GHRM practices, green advocacy behavior increases by 0.065 units for high-green-value employees through the mediation of positive emotions. While this represents a small incremental change for any single individual, its theoretical importance lies in validating the proposed psychological mechanism. It demonstrates that for a specific, targeted subpopulation—employees who already value green activities—GHRM can successfully trigger a positive emotional response that translates into tangible advocacy, however small the initial step may be. This provides a crucial proof-of-concept for the “virtuous cycle” pathway.

Conversely, the effect for the negative pathway (*b* = 0.104), though also modest, highlights a tangible risk for organizations. This finding suggests that for employees with low green values, the implementation of GHRM is associated with a measurable increase in counterproductive behavior, driven by negative emotions. The meaningfulness of this effect is not necessarily in its large impact on a single employee’s behavior, but in its potential cumulative and symbolic consequences. Widespread across an organization, even small increases in cheating behavior can aggregate to erode ethical standards and undermine the very goals of GHRM. Furthermore, this effect is critical from a paradox theory perspective, as it empirically captures the unintended negative consequence that can coexist with positive outcomes.

## 5 General discussion

Recent literature in the field of Green Human Resource Management (GHRM) has focused on various aspects of its application within the hotel industry, with studies such as those by Miah et al. (2024) and Alreahi et al. (2023) offering systematic reviews. These studies categorize the key themes of GHRM into four main areas: employee engagement, marketing systems, corporate social responsibility, leadership and management, and organizational behavior and culture. Building upon this growing body of research, our study integrates. The cognitive appraisal of emotion theory within eco-conscious organizational management, we examine the varied emotional responses employees with differing values have towards green initiatives. Our research generated insight beyond what we already know from existing research which predominantly focused on how GHRM brings favorable effects to the organization and employees (Darvishmotevali and Altinay, 2022). Those with a stronger commitment to green values are more inclined to participate in environmental advocacy, motivated by an upsurge in positive emotions. Conversely, individuals with lower green values tend to experience increased negative emotions towards GHRM, potentially leading to unethical behavior at work. By weaving cognitive appraisal theory into the study of green human resource management, our research introduces several significant implications.

### 5.1 Theoretical implications

Initially, our study expands the current knowledge of GHRM in the hospitality sector by exploring the varied effects arising from different employee behaviors. In recent years, the concept of corporate green management has emerged as a significant area of research. The majority of studies in this field have examined the relationship between Green Human Resource Management (GHRM) and positive employee behaviors, such as pro-environmental behavior (Luu, 2023), environmental passion (Karatepe et al., 2022), and green creativity (Luu, 2023). Responding to the call by Dumont et al. (2017) to explore the broader consequences of GHRM beyond its green outcomes, some studies have found that the implementation of GHRM increases employees’ job satisfaction, work engagement, and reduces turnover intentions (Shafaei et al., 2020; Karatepe et al., 2022). However, these studies predominantly focus on the positive effects of GHRM. To date, insufficient attention has been given to the potential dual-edged effects of GHRM on employee behavior, particularly its potential negative consequences. Our investigation into these uncharted adverse consequences enriches the conversation and provides new insights for companies and their management strategies.

Secondly, our research adds a fresh perspective to GHRM studies by integrating the cognitive appraisal theory of emotion. This approach highlights a new psychological process, demonstrating how individual evaluations and emotions can influence reactions to GHRM efforts in the workplace. The predominant body of research on Green Human Resource Management (GHRM) examines its impact from an organizational perspective, highlighting how GHRM enhances organizational sustainability, financial performance, and reputation through mechanisms such as the resource-based view and employee commitment (Amrutha and Geetha, 2020). A significant portion of the literature also investigates the effect of GHRM through the lens of employees’ internal states, such as learning, motivation, and social influence (Tanova and Bayighomog, 2022). In this study, we explore the relationship between GHRM and employees’ evaluation of these practices from an individual appraisal perspective. By integrating insights from existing literature, we examine how GHRM influences employee emotions and its broader implications on employee perception and engagement. By doing so, we responds to calls for a deeper comprehension of its impacts at the individual level (Arieli et al., 2020; Yong et al., 2020).

Thirdly, by focusing on the hospitality industry from the employees’ viewpoint, our study sheds light on how individual characteristics can lead to paradox responses to GHRM, as suggested by Tandon et al., 2023. We explore the link between individual values and diverse workplace behaviors in the context of GHRM, contributing to the increasing academic interest in how personal differences affect organizational behavior (e.g., Tasselli et al., 2018). Our research emphasizes the crucial role of individual differences in shaping employees’ reactions to GHRM, revealing the complex interplay between green practices and contrasting behaviors. By highlighting the significance of personal values in workplace behavior and responding to the call for a deeper understanding of GHRM from the perspective of individual characteristics (Bahuguna et al., 2023). Additionally, our research suggests that organizations should shift from merely enforcing compliance to actively engaging employees in green objectives, examining how diverse personal values influence their involvement with GHRM initiatives and providing strategies for organizations aiming to align environmental goals with employee development and motivation.

### 5.2 Practical contributions

Key practical contributions of this research include addresses potential adverse consequences, such as increased workplace cheating behaviors driven by negative emotions in individuals with lower green values. For hospitality organizations, it’s essential to enforce strict policies against such behaviors while supporting those who struggle with green changes. Establishing open forums for employees to express their concerns about green initiatives can help mitigate misunderstandings or resistance. This transparent communication is crucial for maintaining a positive work environment and ensuring successful GHRM implementation.

Furthermore, our study underscores the significance of considering employees’ personal values during the implementation of Green Human Resource Management (GHRM) practices. Identifying which employee groups are more inclined to engage in both positive and negative behaviors related to GHRM is essential. For instance, strategies designed to enhance employees’ green values, such as the establishment of specific policies or the promotion of a stronger organizational green climate, can encourage pro-environmental behaviors. Additionally, attention should be paid to fostering individual green values, which can drive employees’ proactive involvement in sustainable practices. As the adoption of green management practices expands within organizations, it is critical for managers to recognize potential shifts in employee attitudes and behaviors and to develop tailored strategies to address these changes effectively. To this end, it is recommended that managers conduct regular one-on-one meetings with employees to assess their concerns and emotions. This can include the use of pulse surveys or informal check-ins to better understand and monitor employee morale.

Considering the diversity inherent in the hospitality industry’s workforce presents unique challenges and opportunities in the implementation of GHRM. Not all employees may accept GHRM initiatives equally, especially those less environmentally inclined. To effectively implement GHRM in this varied setting, managers need inclusive and adaptable strategies. Engaging in dialogue to understand employees’ sustainability views is crucial for designing enriching GHRM strategies. Tailoring these to different cultural contexts ensures better communication and adoption of sustainability principles. In addition, regular environmental training and workshops are essential for cultivating a sustainable culture. For employees with strong green values, specialized training can enhance their advocacy, while incentives for participating in green initiatives can create a positive feedback loop. These sessions should both educate and inspire, emphasizing the environmental impact of actions and their role in the company’s sustainability goals.

Finally, anticipating challenges and preparing contingency plans are crucial for GHRM risk management in hospitality. Proactively identifying potential issues and establishing effective monitoring and feedback mechanisms are necessary to assess the impact of GHRM on environmental goals and employee satisfaction. Regularly seeking employee feedback can offer insights into the effectiveness of GHRM and areas for improvement. For instance, maintaining an open dialogue with employees regarding green practice policies and making necessary adjustments—such as revising reward systems or scheduling green meetings—can enhance engagement. Additionally, given the potential influence of emotional contagion among employees, it is important for management to consider the psychological effects of GHRM initiatives. Recognizing and addressing any negative emotions that may arise is critical to preventing detrimental impacts on organizational performance. Suggest specific programs, such as employee well-being workshops, counseling services, or stress management training, which can help mitigate negative emotions.

## 6 Limitation and future research

Several limitations within this study warrant attention. The primary concern is sample bias, as all participants were from China. Employees of various nationalities may hold differing views and reactions to GHRM, suggesting a need for broader cultural representation in future research (Tanova and Bayighomog, 2022). For example, the concept of power distance, which varies between individualistic and collectivistic cultures, significantly influences human resource management processes and employee behaviors (Daniels and Greguras, 2014). Employees from collectivist cultures with high power distance might exhibit lower propensity to speak up compared to those from individualistic cultures. Secondly, the survey’s methodological constraints necessitate future studies to employ a mixed-method approach for a more holistic understanding of GHRM’s implementation and its impacts. Furthermore, our study is subject to limitations stemming from its sampling and analytical strategies. The sampling method is a concern; although logistically practical, the quasi-random approach involving convenience replacements for non-respondents likely introduced selection bias. This limits the generalizability of our results, as the sample may over-represent certain employee types (e.g., those more readily available or compliant) and under-represent others. Analytically, the dichotomization of personal green values via a ± 1 SD split, though conceptually justified for comparing extreme groups, constitutes a statistical limitation. This choice simplifies interpretation but sacrifices nuanced information and statistical power, potentially obscuring more complex relationships that exist across the full continuum of the variable.

Building on this research, subsequent studies should investigate the varying perceptions employees have towards GHRM programs. Phenomena like “greenwashing” and perceived organizational hypocrisy, where companies superficially adopt green practices for reputation rather than actual environmental commitment (de Freitas Netto et al., 2020; Lauriano et al., 2021), might lead to employees finding less meaning in their company’s GHRM efforts. An employee’s appraisal process, viewed through attribution theory, can provide deeper insights into how organizational hypocrisy influences perceptions of GHRM. Additionally, while our study has identified emotional moderation as a mechanism through which GHRM can have negative effects, future research should uncover other mechanisms that might lead to adverse outcomes. Investigating from a social comparison perspective, for example, could reveal how employees with low green values perceive and react to those with high green values, potentially leading to other consequences like coworker conflict and workplace isolation. Lastly, as GHRM gains traction globally, particularly in the hotel industry, it’s important to consider how employees perceive the absence of green practices in their organizations (Hericher and Bridoux, 2023). This is especially pertinent for those with stronger green values.

## Data Availability

The raw data supporting the conclusions of this article will be made available by the authors, without undue reservation.
